# Silicea in Affections of the Eustachian Tube

**Published:** 1895-06

**Authors:** U. Goullon

**Affiliations:** Weimar


### SILICEA IN AFFECTIONS OF THE EUSTACHIAN TUBE.

DR. U. GOULLON, WEIMAR.

Judge N. complains of troublesome noises in the ear, especially a continuous surging, caused by cardiac irritation without any organic trouble of the heart; he often has a congested face.

He received Silicea, and shortly afterward he returned, astonished that every powder produced, an hour after taking it, a tingling sensation in the nose, extending itself through the Eustachian tube into the internal ear, as if an air-bladder was pushed through and burst. This repeated itself after every dose, and only gradually disappeared. Another patient complained, after having passed through an attack of influenza, of roaring in the ears (it is very hard to get from a patient a clear description of the noises in the ear), and as he complained simultaneously of a recent catarrhal condition of the fauces and nose, it was considered that the Eustachian tube was also affected. He took Silicea for three days, and the noise was gone—*A. H. Z.*, 21, 91. S. L.

